# Large-cell neuroendocrine carcinoma of lung with epidermal growth factor receptor (EGFR) gene mutation and co-expression of adenocarcinoma markers: a case report and review of the literature

**DOI:** 10.1186/2049-6958-8-47

**Published:** 2013-07-18

**Authors:** Yasuhiro Sakai, Takashi Yamasaki, Yoshito Kusakabe, Daisuke Kasai, Yoshikazu Kotani, Yoshihiro Nishimura, Tomoo Itoh

**Affiliations:** 1 Department of Diagnostic Pathology, Kobe University Hospital, 7-5-2 Kusunoki-cho, Chuo-ku, Kobe-shi, Hyogo 650-0017, Japan; 2 Department of Respiratory Medicine, Kobe University Hospital, 7-5-2 Kusunoki-cho, Chuo-ku, Kobe-shi, Hyogo 650-0017, Japan

**Keywords:** Epidermal growth factor receptor, Gene mutation, Large-cell neuroendocrine carcinoma, Lung cancer, Small-cell carcinoma, Tyrosine kinase inhibitor

## Abstract

**Purpose:**

A high rate of response to treatment with epidermal growth factor receptor tyrosine kinase inhibitor (EGFR-TKI) has been observed in certain patients (women, of East Asian ethnicity, with non-smoking history and adenocarcinoma histology) with mutations in exons 18 to 21 of the tyrosine kinase domain of EGFR. Some cases of high-grade neuroendocrine carcinoma of the lung harboring mutations have been sporadically reported.

**Methods:**

We describe the case of a 78-year-old woman with large-cell neuroendocrine carcinoma of the lung, with mutation in exon 21 L858R and co-expression of adenocarcinoma markers.

**Results:**

A mass (3.0 cm in diameter) was identified in the inferior lobe of the left lung, accompanied by metastases into ipsilateral mediastinal lymph nodes and elevations of serum pro-gastrin-releasing peptide and carcinoembryonic antigen. Initial transbronchial brushing cytology suggested high-grade neuroendocrine carcinoma favoring small-cell carcinoma in poorly smeared and degenerated preparations, and revealed exon 21 L858R mutation. Re-enlargement of the cancer and bone metastases was observed after chemotherapy, and further testing suggested large-cell neuroendocrine carcinoma with immunoreactivity to markers of primary lung adenocarcinoma and L858R mutation. High-grade neuroendocrine carcinoma with mutations in the tyrosine kinase domain of EGFR may be associated with adenocarcinoma, as reviewed from the literature and may also apply to our case.

**Conclusions:**

EGFR-TKI could provide better quality of life and survival in patients with advanced or relapsed high-grade neuroendocrine carcinoma with EGFR gene mutations. Further studies in this respect are warranted.

## Background

The entry of tyrosine kinase inhibitors (TKIs) gefitinib (Iressa^®^, AstraZeneca, Wilmington, Delaware) and erlotinib (Tarceva^®^, Genentech, South San Francisco, California), which target epidermal growth factor receptor (EGFR), is one of the most recent, gratifying events in the treatment of advanced non-small-cell lung cancer (NSCLC). Clinical trials have revealed significant variability in response to EGFR-TKIs, and patient characteristics such as sex, dominantly female, East Asian ethnicity, non-smoking history, and adenocarcinoma (ADC) histology have been associated with an increased likelihood of EGFR-TKI effectiveness [1-7]. Furthermore, a high response rate (60 to 90%) to treatment with EGFR-TKIs has been observed in patients harboring mutations in exons 18 to 21 of the tyrosine kinase domain of EGFR, with exon 19 deletions and exon 21 L858R point mutations comprising about 90% of all mutations [8,9]. Although the mechanism of lethal interstitial pneumonia as a side effect of EGFR-TKI is still unknown, EGFR-TKI can be striking in cancer reduction and quality of life improvement in patients with advanced NSCLC harboring EGFR gene mutations. Currently, EGFR-TKI is considered third-line chemotherapy for patients with inoperable and recurrent NSCLC after first-line platinum-based combination chemotherapy and second-line chemotherapy with docetaxel; however, in future the combination of cytotoxic agents and EGFR-TKI may become first- or second-line standard chemotherapy. According to the statement of the International Association for the Study of Lung Cancer/American Thoracic Society/European Respiratory Society about lung ADC [10], EGFR gene mutation should be routinely examined in all patients with NSCLC before nonsurgical treatment and after the initiation of EGFR-TKI therapy, with a view of predicting reactivity and resistance to EGFR-TKI, if possible, with the use of Kirsten rat sarcoma virus oncogene homolog (KRAS) mutation and anaplastic lymphoma kinase (ALK) rearrangement [11-16].

Interestingly, case reports of small-cell lung carcinoma (SCLC) harboring EGFR gene mutation and apparently responding to EGFR-TKI have sporadically appeared since 2005 [17-27]. The mechanism by which SCLC acquires EGFR gene mutation is still unknown, but such cases may occur in association with ADC. Moreover, a few cases of large-cell neuroendocrine carcinoma (LCNEC) with EGFR gene mutations have recently been identified [28-30], an additional case of which is described here.

## Case presentation

**Figure 1:**
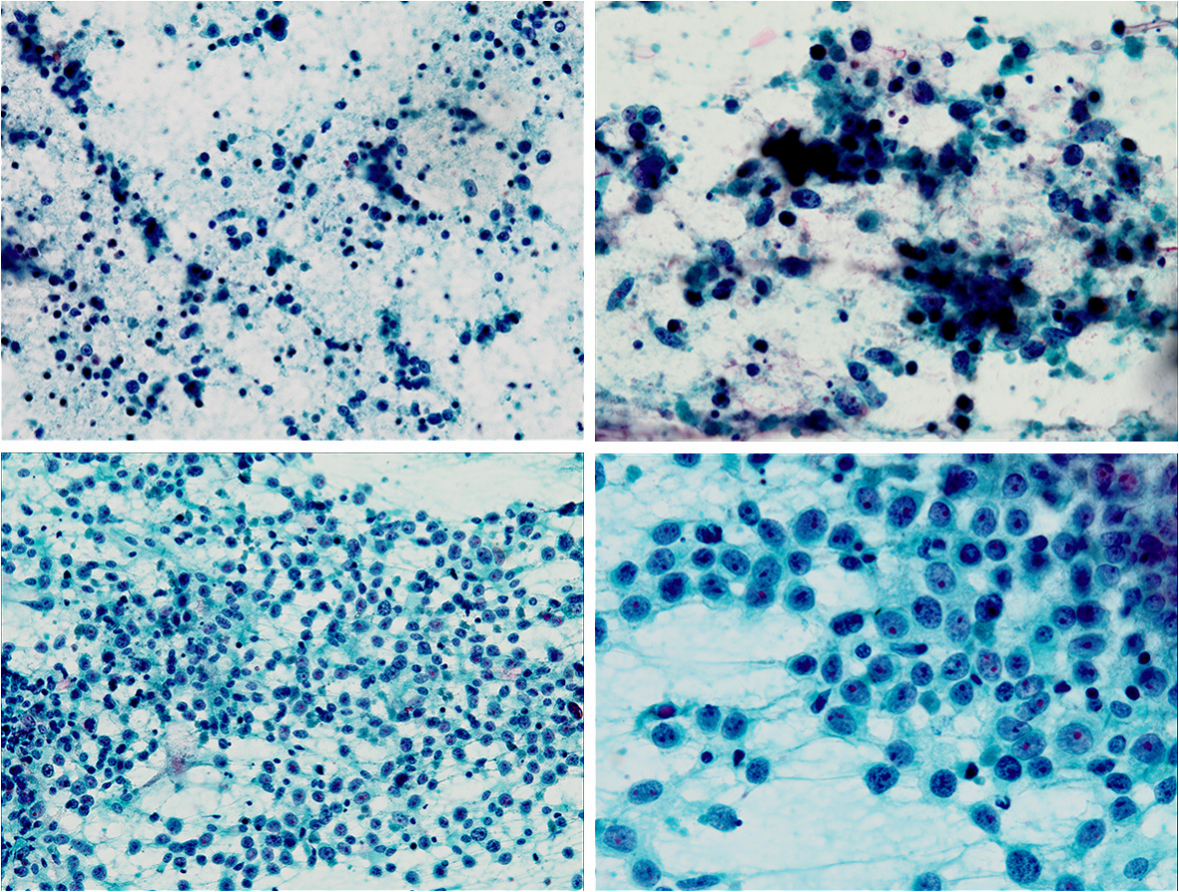
Papanicolaou smears from the first examination diagnosed as high-grade neuroendocrine carcinoma favoring small-cell carcinoma (upper panels left to right: ×200, ×400), and the second examination diagnosed as large-cell neuroendocrine carcinoma (lower panels: ×200, ×400).

**Figure 2:**
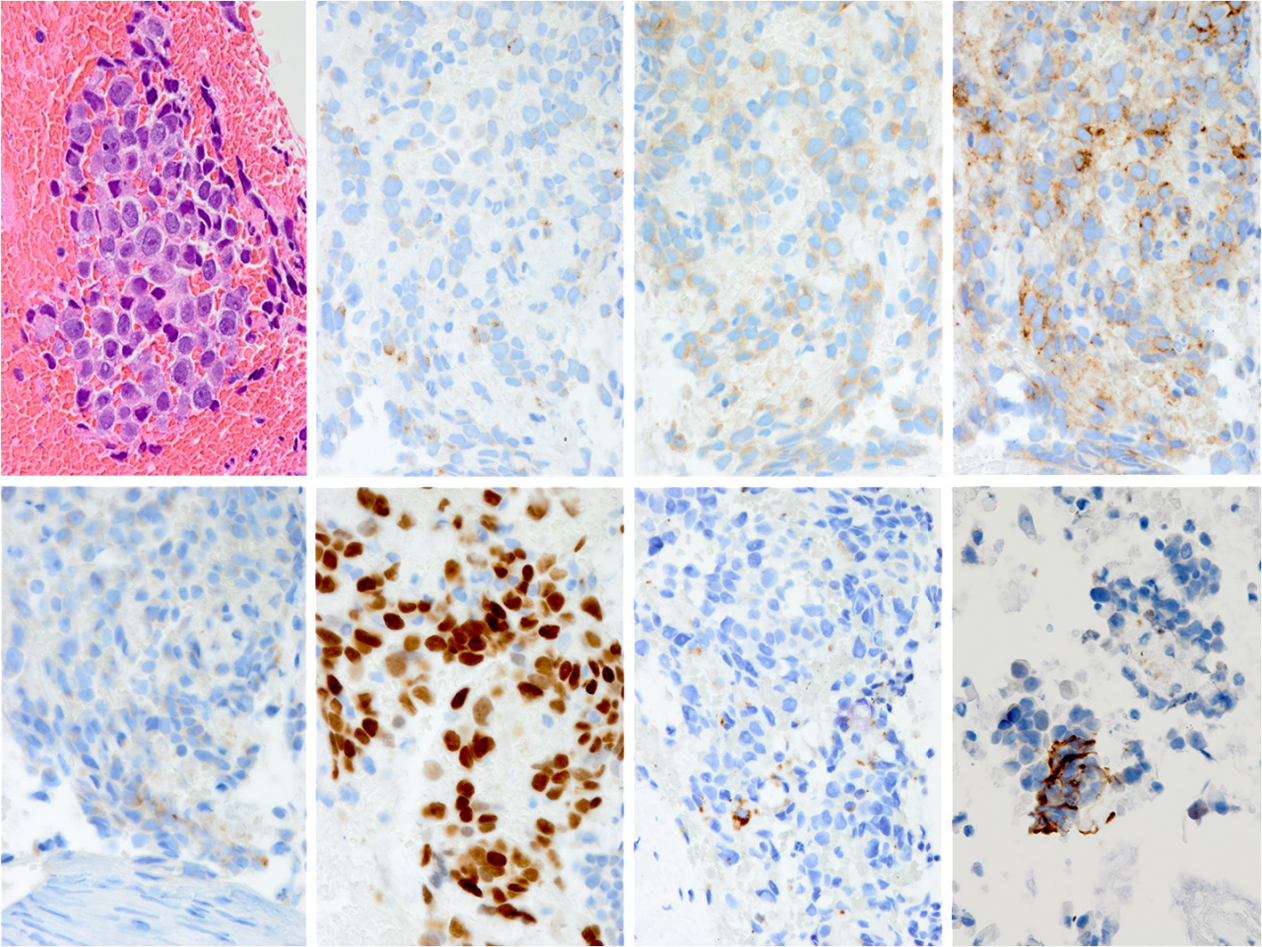
**Histopathologic stains from the second transbronchial biopsy. **Upper panels left to right: hematoxylin and eosin, chromogranin A, synaptophysin, CD56; lower panels: carcinoembryonic antigen, thyroid transcription factor-1, Napsin A, epidermal growth factor receptor L858R mutation (×400).

A 78-year-old Japanese woman ex-smoker (half a pack per day) with past histories of pulmonary tuberculosis and uterine leiomyoma had been under medical treatment for chronic heart failure with atrial fibrillation, unstable angina, eosinophilic myocarditis, bronchial asthma, hyperuricemia, hyperlipidemia, and hypothyroidism. At a follow-up examination one and half year earlier, chest computed tomography showed a mass (1.5 cm in diameter) in the inferior lobe of the left lung. It had doubled in size within the following four months, and positron emission tomography (PET) and magnetic resonance imaging (MRI) revealed metastases to ipsilateral mediastinal lymph nodes. Serum tumor markers were as follows: pro-gastrin-releasing peptide 105 pg/ml (standard range 0 – 80), carcinoembryonic antigen (CEA) 21.8 ng/ml (0 – 5.2), and cytokeratin 19 fragment 3.0 ng/ml (0 – 2). Transbronchial brushing and needle aspiration against the tumor demonstrated small cells with a high nuclear/cytoplasm ratio and fine granular nuclear chromatin scattered around a large cell cluster in poorly smeared Papanicolaou stains, suggesting high-grade neuroendocrine carcinoma favoring small-cell carcinoma (Figure 1, upper panels). EGFR L858R mutation was detected in exon 21 (PNA LNA PCR-Clamp method) of another brushing and aspiration specimen. The patient received four cycles of systemic chemotherapy with carboplatin (area under curve 5) and etoposide (80 mg/m^2^) for three months, at the end of which the size of the tumor decreased by one half. Lumbago developed after six months, and metastases to the left iliac bone and the femur were detected by MRI. Focal radiotherapy (39Gy/13Fr) was carried out. The primary lesion had doubled again during the following four months with enlargement of mediastinal lymph nodes. Histopathologic and cytologic follow up examinations were carried out: Papanicolaou smears revealed cells larger than those at previous examinations, and numerous scattered sheet-like arrangements. The cells had fine granular nuclear chromatin and prominent large nucleoli of a high nuclear/cytoplasm ratio and relatively rich cytoplasm (Figure 1, lower panels). Transbronchial lung biopsy failed to provide detailed histology of the tumor cells because of insufficient sampling volume, and proliferative patterns such as organoid structures and nuclear molding could not be identified. Immunohistochemical analysis showed tumor cell immunoreactivity to three neuroendocrine markers: chromogranin A, synaptophysin, and CD56 (Figure 2). Retrospectively, the cells subject of the second examination showed almost the same morphology as that of cells of the first test regardless of artifactual degeneration. Consequently, the tumor was diagnosed as large-cell neuroendocrine carcinoma. Thyroid transcription factor-1 was diffusely and strongly positive in the cells. Interestingly, CEA and Napsin A were also focally positive. It is noteworthy that the mutation-specific immunostaining for the detection of EGFR exon 21 L858R mutation (rabbit monoclonal antibody, 1:100, clone 43B2, Cell Signaling Technology^®^, Danvers, MA) was positive (Figure 2). Second-line chemotherapy is under consideration at present.

## Discussion

With the present case we aim to demonstrate a possible additional case of LCNEC with EGFR gene mutation. Three other cases have been reported retrospectively (Table 1), and although no clinicopathologic feature has been identified in them, two have occurred in non-smokers, two show EGFR gene exon 19 deletion mutation, and one demonstrates exon 18 point mutation [28-30]. Ours may be the first in an ex-smoker with LCNEC harboring exon 21 L858R point mutation. The mechanism and significance of EGFR gene mutation in LCNEC is still unknown. Nonetheless, twenty cases of SCLC with EGFR gene mutation have been reported and are used in this discussion (Table 1) [18-27]. Among these SCLC cases, the ratio of male: female is 1:3 (age 60.9 ± 26.1 years; range, 36 – 89), and of smoker: non-smoker 2:3. Strikingly, fourteen (70%) show combined SCLC and adenocarcinoma, or suggest a transformation from adenocarcinoma to SCLC; eleven display EGFR gene exon 19 deletion mutation, and eight exon 21 L858R point mutation proportionally similar to mutations in adenocarcinoma. Whether LCNEC with EGFR gene mutation is related to adenocarcinoma is still unknown, although only one case has suggested transformation from adenocarcinoma to LCNEC [30]. The present case was, however, immunoreactive to adenocarcinoma markers TTF-1, Napsin A, and CEA, not to entrapped non-tumor alveolar cells, which was a conspicuous finding when considering the occurrence of LCNEC with EGFR gene mutation.

**Table 1:** Review of epidermal growth factor receptor gene mutation in patients with high-grade neuroendocrine carcinoma and its combination with other types

**Authors**	**Age**	**Gender**	**Smoking**	**Stage**	**Cell type**	**Specimen**	**Activating EGFR mutation**
Okamoto et al. (2006)	72	F	No	IV	SCLC	biopsy	exon 19, del E746-A750
Zakowski et al. (2006)	45	F	No	IV	ADC → SCLC	biopsy	exon 19, del L747-P753insQ
Morinaga et al. (2007)	46	F	No	IIIB	ADC → SCLC	biopsy	exon 19, del E746-A750
Fukui et al. (2007)	62	F	No	IIIB	combined SCLC and ADC	resection	exon 21, L858R mutaion
Tatematsu et al. (2008)	36	F	No	IV	combined SCLC and ADC	resection	exon 21, L858R mutaion
	81	M	Yes	IV	SCLC	biopsy	exon 18, G719A point mutation
	69	M	Yes	IA	combined SCLC and ADC	biopsy	exon 21, L858R mutaion
	89	F	Yes	IB	SCLC	biopsy	exon 21, L858R mutaion
	65	M	Yes	IIA	combined SCLC and ADC	resection	exon 19, 15-bp deletion
Alam et al. (2010)	73	F	No	IV	ADC (lung), SCLC (liver)	biopsy	exon 21, L858R mutaion
Shiao et al. (2011)	63	M	Yes	III	SCLC	biopsy	exon 19 deletion (del E746-S752 insV)
	54	F	No	II - III	SCLC	biopsy	exon 19 deletion (del E746-A750)
Sequist et al. (2011)	67	F	NA	NA	ADC → SCLC	biopsy	exon 21, L858R mutaion
	54	F	NA	NA	ADC → SCLC	biopsy	exon 19 deletion
	56	F	NA	IV	ADC → SCLC	biopsy	exon 21, L858R mutaion
	40	F	NA	NA	ADC → SCLC	biopsy	exon 19 deletion
	61	F	NA	NA	ADC → SCLC	biopsy	exon 21, L858R mutaion
Iyoda et al. (2011)	NA	NA	NA	NA	LCNEC	resection	exon 18, codon 725 (ACG to ACA)
De Pas et al. (2011)	66	M	No	IV	LCNEC	biopsy	exon 19 deletion (p.L747_A755>AT)
Yanagisawa et al. (2012)	46	M	No	IIIA	ADC → LCNEC	resection → biopsy	exon 19 micro-deletion
Lu et al. (2012)	61	M	Yes	IIA	combined SCLC and SQCC	resection	exon 19, del E746-A750 (K745 AAA)
	62	F	No	IIIA	combined SCLC and ADC	resection	exon 19, del E746-A750 (K745 AAA)
Lu et al. (2012)	62	F	No	IIIA	combined SCLC and ADC	resection	exon 19 mutation, codon 746-754
Our case (2012)	78	F	Yes (Ex)	IV	LCNEC	brushing	exon 21, L858R mutaion

ADC, adenocarcinoma; EGFR, epidermal growth factor receptor, F, female; LCNEC, large-cell neuroendocrine carcinoma; M, male; NA, not available; SCLC, small-cell lung cancer; SQCC, squamous cell carcinoma.

Distinctly, the detection of EGFR exon 21 L858R mutation by PCR on the first specimen might have been due to the very few adenocarcinoma cells, combined type with adenocarcinoma. Adenocarcinoma might have been present before the development of LCNEC and before the first biopsy. It can be assumed that the present case was consistently adenocarcinoma with neuroendocrine differentiation harboring EGFR exon 21 L858R mutation. Neuroendocrine proliferative patterns, such as organoid ones and nuclear molding, were not identified in the whole section of the surgical specimen. Also, the clonal relation between the first and second tests was not clear because the mutational status of the EGFR gene in the second biopsy was not addressed by PCR. Nonetheless, this case might be consistently LCNEC harboring EGFR exon 21 L858R mutation, especially that the cytologic features displayed by both tests were almost the same, noting specially the PCR conducted on the same tumor cells as those of the Papanicolaou preparations, and the positivity of EGFR exon 21 L858R mutation-specific immunostaining in the second biopsy. Indeed, high-grade neuroendocrine carcinoma harboring EGFR gene mutation may have molecular adenocarcinomatous characteristics, at least by derivation from the literature about cases like the present one. Further studies are needed on the occurrence of high-grade neuroendocrine carcinoma harboring EGFR gene mutation.

An additional therapeutic choice for high-grade neuroendocrine carcinoma showing poor prognosis is expected, and EGFR-TKI that is now available commercially should naturally be considered even for this carcinoma, much more than for one with adenocarcinomatous characteristics. Presently there is no regimen of chemotherapy for high-grade neuroendocrine carcinoma harboring EGFR gene mutation. Nonetheless, the detection of this disease needs to be attempted through further study. The prerequisite to administering EGFR-TKI for NSCLC is the presence of EGFR gene mutation, as opposed to resistance mutation such as that of exon 20 T790M, exon 19 D761Y, and exon 21T854A [11,31,32]. Testing for the presence of EGFR gene mutation by PCR in SCLC and LCNEC may, however, be practically difficult from the aspect of cost and rarity. Immunohistochemistry may be one of the strategies for easy detection of EGFR gene mutation. Available and noteworthy are two mutation-specific antibodies for the detection of EGFR gene mutations: anti-EGFR exon 19 E746-750 deletion antibody (rabbit monoclonal, clone 6B6, Cell Signaling Technology^®^, Danvers, MA) along with anti-EGFR exon 21 L858R mutation antibody [33,34]. Anti-EGFR exon 21 L858R mutation-specific immunostaining of lung adenocarcinoma demonstrates excellent sensitivity (95.2%) and specificity (98.8%), although the former may react to exon 19 deletion overlapping E746-A750. Immunostaining with these antibodies is less costly and easier to conduct than by molecular detection of EGFR gene mutation, covers the two major patterns of EGFR gene mutation, and can be used for SCLC and LCNEC in which EGFR gene mutation is less frequent than in ADC.

## Conclusions

The fact that patients with lung cancer have as many therapeutic choices as possible is gratifying. High-grade neuroendocrine carcinoma generally shows more aggressiveness and rapid relapse than NSCLC. Interestingly, our case suggests the presence of high-grade neuroendocrine carcinoma harboring EGFR gene mutation: EGFR-TKI may provide better quality of life and survival in patients with advanced or relapsed high-grade neuroendocrine carcinoma, particularly that with EGFR gene mutation. EGFR gene mutation-specific immunostaining is potentially effective and should be utilized for easy detection and further study of this rare disease.

## Consent

Written informed consent was obtained from the patient for publication of this case report and any accompanying images. A copy of the written consent is available for review by the Editor-in-Chief of this journal.

## Competing interests

The authors have no grant for this research and no competing interest to declare.

## Authors’ contributions

All authors read and approved the final manuscript.
